# Nanomaterials for Biosensing Applications

**DOI:** 10.3390/nano6040058

**Published:** 2016-03-30

**Authors:** Sichao Hou, Aiying Zhang, Ming Su

**Affiliations:** 1 Department of Chemical Engineering, Northeastern University, Boston, MA 02115, USA; hou.si@husky.neu.edu; 2 School of Materials Science and Engineering, Beijing Institute of Technology, Beijing 100081, China; zhay@bit.edu.cn

Nanomaterials have shown tremendous potentials to impact the broad field of biological sensing. Nanomaterials, with extremely small sizes and appropriate surface modifications, allow intimate interaction with target biomolecules. Ever since the pioneering work of Chad Mirkin [1] and others on nanoparticle-based biosensing, a variety of nanostructured materials with unique optical [2], electric [3], magnetic [4], electrochemical [5], and thermal signatures [6] have been used to detect molecular biomarkers with extreme high sensitivities. The sensing mechanism is based on converting target binding events into physical signals that can be amplified and detected. Nanomaterials have been widely used for *in vitro* detection of molecular biomarkers of many diseases (such as cancer, neurodegenerative diseases, and infectious diseases, among others) released into patients’ body fluids during disease progression. Nanomaterials have also been used as imaging contrasts to map out the spatial distribution of biomarkers *in vivo* when combined with an imaging modality such as magnetic resonance imaging (MRI) or computed tomography (CT). Given the importance of biosensing in the biomedical field, it is imperative to update the recent advance in the field of nanomaterial biosensing.

This Special Issue of *Nanomaterials*, “Nanomaterials for Biosensing Applications,” is focused on the synthesis, properties, and prospective biological sensing applications of nanomaterials. The topics cover a wide range of research fields including physical science, engineering, and medicine in the forms of review, communication, and academic articles. The potential use of bimetallic nanoparticles in the biosensing field as the catalytic amplifying materials in the third-generation biosensors is covered in the review by Rick *et al.* [7]. A large portion of the articles in this special issue cover multiple specific applications of nanoparticles in biosensing, distinguished by the target analyte from glucose [8], nucleic acid [9], H_2_O_2_ [10], laccase [11], Cd^2+^, and Pb^2+^ [12] to phosphatase [13]. A theoretical guideline for biosensor designing is reported by Tan *et al.* in a numerical analysis of nanoparticle dispersion in red blood cells [14]. Given the widespread applications, these contributors devote themselves in searching for facile and cost-saving designs of biosensors for varieties of targets and characterizing the response, the sensitivity, and the limitation of nanomaterial-based biosensors.

Starting with a definition of the biosensor—“a device for the detection of an analyte that couples a bio-recognition element to a signal transducer to generate a measurable electrical signal”—the importance of biosensors in providing real-time quantitative information about the composition of the sensor situated environment is illustrated in the review by Rick *et al.* [7]. Three generations of the biosensors design progress are introduced next: the enzyme-dependent biosensor, the mediator-based biosensor, and the catalytic electrode-dependent biosensor, among which the third generation attracts most attention. Third-generation sensors originally attempted to co-immobilize the metallic enzyme and mediator on the surface of an electrode, thus making the bio-recognition component an integral part of the electrode transducer. The metal nanoparticles, introduced into a catalytic structure, potentially afford opportunities for both catalytic fine-tuning and cost-saving. Following this clue, 13 types of target-based biosensors and the synthesis of 3 types of bimetallic nanoparticles are stated. Binary nanostructured materials exhibit clearly different physical and chemical properties compared to their individual components, which confer greater catalytic activity and can be further improved and tailored specifically.

One-dimensional (1D) nanostructures offer direct and fast electron transport between the electrode substrates and enzyme, which significantly improves the sensitivity. However, the small surface area limits the application of 1D nanostructures in biosensors [15]. A three-dimensional (3D) amperometric glucose biosensor was fabricated by layer-by-layer self-assembly of gold nanorods (AuNRs) and glucose oxidase (GOD) onto a single-walled carbon nanotube (SWCNT)-functionalized sol-gel matrix by Wu *et al.* [8]. The sol-SWCNTs-(AuNRs-GOD)_4_/Au biosensor exhibited a linear relation within the range of 0.01-8 mM glucose, a high sensitivity of 1.08 μA/mM, and a fast amperometric response within 4 s. According to the authors, the good performance of this amperometric glucose biosensor is due to the merits of the 3D sol-gel matrix and stereo self-assembly films, and the natural features of one-dimensional nanostructure SWCNTs and AuNRs.

Upconversion nanoparticles (UCNPs) have been used to detect quantum dot (QD)-labeled nucleic acid. However, QD labels retard the kinetics of hybridization due to large spatial resistance; meanwhile, QD labels are costly and toxic, limiting their practical applications [16]. A paper-based sandwich hybridization assay was used to detect unlabeled nucleic acid using UCNPs [9]. The combination of Cy3-labeled reporter DNA and the unlabeled target, which was pre-hybridized with surface-bound probe DNA, provided the proximity between Cy3 and UCNP for the luminescence resonance energy transfer (LRET) process. A ratiometric method was optically stimulated by illuminating the UCNPs under a 980-nm laser. A limit of detection (LOD) of 146.0 fmol was obtained by the proposed assay, with the LRET efficiency attributed to the distance between the donor and acceptor. Selectivity can be tuned by the use of formamide, and a maximum signal ratio (1.81) was achieved between fully complementary target (FCT) and one base pair mismatch (1BPM). The sandwich format hybridization assay remained available even in undiluted serum, which suggests the potential for oligonucleotide determination in clinical origin samples with a complicated background matrix. Thus far, the signal has been collected by a bench-top fluorescent microscope, whereas the authors expect to use smartphone cameras as a replacement, making this assay more portable and low-cost.

As a simple but effective technique, electrospinning has been widely applied to synthesize organized functional polymer nanofibers (NFs), with exceptionally long length, uniform diameter, and large surface area, and enzyme-based electrochemical sensors. However, the non-enzymatic electrochemical biosensors based on electrospun NFs on electrodes have seldom been studied [17]. Nanoporous carbon nanofibers (CNFs) decorated with platinum nanoparticles (PtNPs) using electrospining polyacrylonitrile (PAN) nanofibers and subsequent carbonization and binding of PtNPs was reported by Li *et al.* [10]. The nanoporous CNF-PtNP hybrids were further utilized to modify glass carbon electrodes and for the non-enzymatic amperometric biosensor to detect the hydrogen peroxide (H_2_O_2_) with a limit of detection of 1.9 μM. The cyclic voltammetry experiments and the chronoamperometry measurements indicate that a high electrocatalytic activity and a high sensitivity were obtained for CNF-PtNP modified electrodes and the fabricated biosensors for the detection of H_2_O_2_. This work is not only a guideline for further design and fabrication of nanofiber-based devices, but also extend the possibility in energy storage, cytology, and tissue engineering.

Laccase (Lcs)-based biosensors show two advantages: (i) The presentation of molecular oxygen, the reoxidizing agent, in the solution is self-sufficient for the reaction; and (ii) The capability of Lcs connecting the direct electron transfer (DET) to the transducers allows the construction of mediator-free electrodes [18]. Thus, Lcs was chosen as the model enzyme by Favero *et al.* [11] to investigate the possibility of entrapping gold nanoparticles (AuNPs) onto a support of graphene (GPH) screen-printed electrodes and multi-wall-carbon-nanotube (MWCNT) screen-printed electrodes, which enhance the heterogeneous electron transfer (HET) between Lcs and the electrode support. The characterization results showed that the use of AuNPs to modify both graphene and MWCNT screen-printed electrode surfaces enhanced the electrochemical performances of the electrodes. More satisfying results were obtained for MWCNTs in terms of higher electroactive area formation after the modification. The two modified electrodes were successively proven to efficiently immobilize the trametes versicolor (TvL); the properties of electrochemical sensing for the GPH- and MWCNT-based AuNPs–TvL biosensors were studied by choosing various laccase mediators. The proposed strategy for the modification of the electrode therefore extends significant future applications for biosensors in clinical-medical, pharmaceutical, and environmental fields.

Due to their non-biodegradability and persistence, cadmium(II) and lead(II) are severe hazardous environmental pollutants, which are toxic on living organisms [19]. The widespread methods for heavy metals analysis, including atomic absorption spectrometry (AAS), atomic emission spectrometry (AES), atomic fluorescence spectrometry (AFS), and inductively coupled plasma mass spectrometry (ICP-MS), have limited applications due to the high cost and the difficulty for the on-line analysis. An alternative method, a glassy carbon electrode (GCE) modified with self-doped polyaniline nanofibers (SPAN)/mesoporous carbon nitride (MCN) and bismuth is proposed by Zhang *et al.* [12], through which trace Cd^2+^ and Pb^2+^ could be determined simultaneously by square-wave anodic stripping voltammetry (SWASV). Under the optimum conditions, the fabricated electrode exhibited a linear relation ranging from 5 to 80 nM for each analyte with a limit of detection (LOD) to be 0.7 nM and 0.2 nM for Cd^2+^ and Pb^2+^ (S/N = 3), respectively. Additionally, the electrode exhibited excellent performance on the repeatability, reproducibility, and anti-interference ability. The combination of carbon materials and SPAN in electrode fabrication would offer an alternative route to fabricate new types of electrodes aimed at heavy metals determination.

Phosphorylation and dephosphorylation play important parts in cellular regulation and signaling processes, including metabolism, gene transcription and translation, cytoskeletal rearrangement, and apoptosis [20]. A hypothesis was built by Sun *et al.* [13] that the quenching efficiency of graphene oxide (GO) to the phosphorylated and dephosphorylated dye-labeled peptides could be distinguished by tuning the interaction between the phosphate group and the amino residue. Based on this hypothesis, an approach for fabricating GO-based fluorescent biosensors to survey the variation of phosphorylation state and phosphatase activity is presented. Alkaline phosphatase (ALP) was tested as a model enzyme with a probe of phosphorylated fluorescein isothiocyanate (FITC)-labeled short peptide FITC–Gly–Gly–Gly–Tyr(PO32-)–Arg. When the negatively charged phosphate group in the Tyr residue was removed from the peptide substrate via enzymatic hydrolysis, the electrostatic interaction resulted in the adsorption of FITC–Gly–Gly–Gly–Tyr–Arg onto the GO surface. With the rational design of the substrate sequence, the authors believe that this concept can be inspiring for the development of other GO-based fluorescent biosensors. Further, through using a multi-well plate reader, the method will be valuable for the screening of new enzyme inhibitors and drugs in a high-throughput layout.

Besides experimental works, theoretical analysis can also be found in this special issue. Under the fact that there is no analytical formula or quantitative rule to directly predict the nanoparticle (NP) dispersion rate, which is much needed in large-scale drug delivery simulations, Tan *et al.* [14] presents a numerical study on NP transport and dispersion in red blood cell (RBC) suspensions under shear and channel flow conditions, which aimed at obtaining an empirical formula to predict NP dispersion rate within a range of shear rates and cell concentrations. Compared to the observations from previous experimental and numerical studies, the proposed formula showed an accurate prediction on the NP dispersion rate, which is strongly influenced by local disturbances resulted from the motion and deformation in the RBC. The proposed method provides an efficient tool for estimating the NP dispersion rate in modeling NP transport in large-scale vascular networks without explicit RBC and NP models.

In summary, this special issue collects a compilation of articles that prominently demonstrate the continuous efforts in developing novel nanomaterial-based biosensors for various targets, as well as the enormous potentials provided by these biosensors.
